# UTILITY OF EXECUTIVE FUNCTION TO IDENTIFY EARLY COGNITIVE IMPAIRMENT IN AFRICAN AMERICANS

**DOI:** 10.1093/geroni/igz038.048

**Published:** 2019-11-08

**Authors:** Stephanie Garrett, Felicia C Goldstein, Yunyun Chen, Kirk Easley, Darius McDaniel, Janice Lea, Tiffany Thomas, Sabria Saleh, Ihab Hajjar

**Affiliations:** Emory University, Atlanta, Georgia, United States

## Abstract

Within diverse cohorts, African Americans (AA) demonstrate higher rates of Alzheimer’s dementia (AD) and Alzheimer’s dementia combined with multiple comorbid conditions. AA are also two times more likely to develop late-onset AD than whites and less likely to be diagnosed. Yet, our understanding of this disparity in cognition remains limited. Cognitive impairment (CI) and dementia are both underdiagnosed and underreported in primary care patients. The lack of early detection of cognitive and functional decline in high risk populations results in failure to provide care and interventions to members of vulnerable groups. This talk will focus on research that seeks to identify a more sensitive cognitive marker for early identification of cognitive impairment for AA and advances this objective by linking the cognitive marker to AD cerebral spinal fluid biomarkers (Aβ1-42, p-tau) and testing whether this association differs between AA and whites.

